# Negative pressure pulmonary edema - a life-threatening condition in an eye care setting: a case report

**DOI:** 10.1186/s13256-016-0820-z

**Published:** 2016-02-24

**Authors:** Ramesh Venkatesh, Preety Gautam, Parul Dutta, Preeti Bala

**Affiliations:** Dr. Shroff’s Charity Eye Hospital, 5027, Kedarnath Road, Daryaganj, New Delhi, 110002 India

**Keywords:** Negative pressure pulmonary edema, General anesthesia, Complication, Eye surgery

## Abstract

**Background:**

Negative pressure pulmonary edema is a potentially life-threatening complication after general anesthesia in young healthy individuals that results from upper airway obstruction followed by strong inspiratory effort. It is a known complication after nasal or upper airway surgery. Occurrence of such a life-threatening complication in an eye care setting where advanced intensive care is usually lacking is rare.

**Case presentation:**

A 15-year-old Asian boy presented to our hospital with a penetrating eye injury caused by a pellet. Globe and vitreoretinal exploratory surgery was performed with the patient under general anesthesia. The patient’s postoperative course was uneventful immediately after the procedure, but soon he developed negative pressure pulmonary edema.

**Conclusions:**

This case report highlights the importance of early diagnosis and prompt management of negative pressure pulmonary edema to save the life of the patient. Most ophthalmologic surgeries are performed with the patient under regional anesthesia; very few are done with the patient under general anesthesia. Intensive care facilities are needed in such settings for prompt management of such a serious and rare complication.

## Background

Negative pressure pulmonary edema (NPPE) is a potentially life-threatening complication following general anesthesia (GA) in young healthy individuals. It is a manifestation of upper airway obstruction (UAO), followed by strong inspiratory effort, in healthy individuals during recovery from GA. NPPE is a known complication after nasal or upper airway surgery [1, 2]; however, its occurrence after ocular surgery is rare. We report one such case of NPPE following vitreoretinal surgery at a tertiary eye institution where advanced lifesaving measures were lacking.

## Case presentation

A tall, 70-kg, 15-year-old Asian boy presented to our hospital for eye and vitreoretinal exploratory surgery under GA. He was in American Society of Anesthesiologists grade I with uncomplicated grade I intubation, and his blood investigations and chest x-ray were within normal limits. He had no past history of nasopharyngeal surgeries. In the preoperative area, the patient was premedicated with injectable ondansetron 0.1 mg/kg, injectable glycopyrrolate 10 μg/kg, injectable midazolam 0.05 mg/kg, and injectable fentanyl citrate 2 μg/kg. Intraoperative monitoring included electrocardiography, blood pressure monitoring, blood oxygen saturation by pulse oximetry (SpO_2_), and end-tidal capnography.

The patient was preoxygenated with 100 % oxygen through a face mask for 3 minutes. Anesthesia was induced with injectable propofol 2 mg/kg, and then intubation was performed with intravenous vecuronium 0.1 mg/kg. Anesthesia was maintained with oxygen and nitrous oxide 0.5 % in 1 % isoflurane. The perioperative period was uneventful. Upon completion of the procedure, the effect of the relaxant was reversed using neostigmine 2.5 mg and glycopyrrolate 0.5 mg administered intravenously. The patient was then extubated, which was uneventful, and an adequate recovery was achieved.

It was decided to shift the patient to the postoperative ward, as he was fully awake. While shifting, he suddenly developed a brief episode of laryngospasm and respiratory distress in the form of paradoxical chest movements. His SpO_2_ suddenly dropped to 40 %, and his skin became dusky. He was unable to breathe due to UAO. Mask ventilation with 100 % oxygen was attempted but not possible, as the patient started desaturating further. Injectable suxamethonium chloride 50 mg was given intravenously, followed by bag mask ventilation. The patient’s saturation started improving. While he was fully conscious and responding to commands, pink, frothy sputum was noticed suddenly and bilateral crepitations were present.

A probable diagnosis of NPPE was made. The patient was shifted to an intensive care unit in another hospital. One hundred percent continuous positive airway pressure was maintained with bag valve mask and injectable furosemide 40 mg, along with maintenance parenteral fluids. The patient’s status returned to normal by the next morning.

## Discussion

NPPE was first hypothesized in 1927 by Morre but was first described by Oswalt in 1977 [1]. It is an often misdiagnosed or less thought of entity occurring during or after recovery from GA. The incidence of NPPE has been reported to be 0.05–0.1 % of all anesthetic practices; however, it is suggested to occur more commonly than is generally documented [1].

NPPE is defined as a form of noncardiogenic pulmonary edema that results from the generation of high negative intrathoracic pressure following spontaneous breathing against UAO. NPPE is a potentially life-threatening complication occurring in apparently healthy young individuals with athletic builds. Any and every case of UAO can lead to NPPE [1]. Laryngospasm during intubation or during recovery after anesthesia is the most commonly reported etiology of NPPE in adults [2].

Risk factors enumerated in causation of NPPE include obese individuals in whom intubation is difficult; presence of any airway lesions; history of nasal, oral, or pharyngeal surgery; young, athletic males; and pediatric patients. Our patient had a well-built athletic body. This was his only risk factor for NPPE. His intubation was uncomplicated, and he had no UAO.

The most common cause of postobstructive pulmonary edema is laryngospasm during intubation or after anesthesia in the postoperative period [3]. Laryngospasm has been reported to be the cause in more than 50 % of cases of postobstructive pulmonary edema [1–3]. The most probable cause in our patient was laryngospasm.

Our patient developed tachypnea; pink, frothy secretions; and rapidly decreasing oxygen saturation. These signs are consistent with a diagnosis of NPPE as described by Halow and colleagues [4] and Goldenberg and coworkers [5]. The differential diagnosis includes aspiration of gastric contents, acute respiratory distress syndrome, volume overload, anaphylaxis, and airway obstruction.

NPPE otherwise is a self-limiting condition. The mainstay of treatment is usually supportive, with administration of diuretics or reintubation, if necessary. Our patient responded well to diuretics and supplemental oxygenation.

## Conclusions

NPPE should be kept in mind as a probable diagnosis in any and every case of UAO. Prompt diagnosis is very important to limit morbidity and mortality in such cases. Uncomplicated ocular surgeries can be life-threatening as well. This case report also stresses the need for lifesaving facilities in ophthalmologic institutions where very few ocular surgeries are performed with patients under GA.

## Consent

Written informed consent was obtained from the patient’s legal guardian(s) for publication of this case report and any accompanying images. A copy of the written consent is available for review by the Editor-in-Chief of this journal.

## Abbreviations

GA: general anesthesia
NPPE: negative pressure pulmonary edema
SpO_2_: blood oxygen saturation by pulse oximetry
UAO: upper airway obstruction
